# Oral biofilm composition and phenotype in caries-active and caries-free children

**DOI:** 10.3389/froh.2024.1475361

**Published:** 2024-10-22

**Authors:** Gabriella Boisen, Susanne Brogårdh-Roth, Jessica Neilands, Alex Mira, Miguel Carda-Diéguez, Julia R. Davies

**Affiliations:** ^1^ Section for Oral Biology and Pathology, Faculty of Odontology, Malmö University, Malmö, Sweden; ^2^ Biofilms Research Center for Biointerfaces, Malmö University, Malmö, Sweden; ^3^ Department of Paediatric Dentistry, Section 4, Faculty of Odontology, Malmö University, Malmö, Sweden; ^4^ Department of Health and Genomics, Foundation for the Promotion of Health and Biomedical Research (FISABIO), Valencia, Spain; ^5^ School of Health and Welfare, University of Jönköping, Jönköping, Sweden

**Keywords:** oral biofilm, dental caries, microbiome, acid tolerance, metabolomics, NMR

## Abstract

**Introduction:**

During development of dental caries, oral biofilms undergo changes in microbial composition and phenotypical traits. The aim of this study was to compare the acid tolerance (AT) of plaque from two groups of children: one with severe caries (CA) and one with no caries experience (CF) and to correlate this to the microbial composition and metabolic profile of the biofilms.

**Methods:**

Dental plaque samples from 20 children (2–5 years) in each group were studied. The AT was analyzed by viability assessment after exposure to an acid challenge (pH 3.5), using LIVE/DEAD® BacLight™ stain and confocal microscopy. Levels of acid tolerance (AT) were evaluated using a scoring system ranging from 1 (no/low AT), to 5 (high/all AT). Metabolic profiles were investigated following a 20 mM glucose pulse for one hour through Nuclear Magnetic Resonance (NMR). Microbial composition was characterized by 16S *r*RNA Illumina sequencing.

**Results:**

The mean AT score of the CA group (4.1) was significantly higher than that of the CF group (2.6, *p* < 0.05). When comparing the end-products of glucose metabolism detected after a glucose-pulse, the CA samples showed a significantly higher lactate to acetate, lactate to formate, lactate to succinate and lactate to ethanol ratio than the CF samples (*p* < 0.05). The bacterial characterization of the samples revealed 25 species significantly more abundant in the CA samples, including species of *Streptococcus, Prevotella, Leptotrichia* and *Veillonella* (*p* < 0.05)*.*

**Discussion:**

Our results show that AT in pooled plaque from the oral cavity of children with severe caries is increased compared to that in healthy subjects and that this can be related to differences in the metabolic activity and microbial composition of the biofilms. Thus, the overall *phenotype* of dental plaque appears to be a promising indicator of the caries status of individuals. However, longitudinal studies investigating how the AT changes over time in relation to caries development are needed before plaque AT could be considered as a prediction method for the development of dental caries.

## 1 Introduction

Dental caries is a highly prevalent disease which affects approximately 35% of the world population and caries in the primary dentition of children under the age of six, is the 12th most prevalent condition globally, with a considerable impact on the child´s wellbeing (1–3). In addition, caries early in life is a predictor for continued disease later in life (4).

Caries development is induced by the metabolic activity of functionally organized, multi-species biofilms consisting of various microorganisms such as bacteria, fungi, archaea and viruses. The community structure of the biofilm provides protection of the species present as well as facilitating horizontal gene-transfer, cell-cell signaling and nutritional cooperation. The interactions taking place in these biofilms result in microbial properties not discernible in studies on single strains (5). Furthermore, biofilm formation induces phenotypical changes in the organisms present, such as increased resilience to environmental stressors (6, 7).

In oral health there is a dynamic stability between the biofilm and the host, with the pH of the oral environment fluctuating around neutral. The microbial metabolism of carbohydrates results in the production of organic acids, where the type and the metabolic pathway used is dependent on the specific species or strain (8). During caries development there is a local increase in organic acids in the biofilm – either through impaired buffering capacity of the host saliva or increased carbohydrate metabolism, or both. This leads to a decrease in biofilm pH which eventually results in demineralization of dental hard tissues as well as an ecological shift in the biofilm, where microbes with a higher tolerance to an acidic environment are selected and increase in abundance (9). Thus, according to this model, a phenotypic shift towards an increase in the overall acid tolerance (AT) of the biofilm would occur at the early stages in the development of a caries lesion.

A lower species diversity is often observed in plaque samples of caries-active individuals compared to those who are caries-free, suggesting that specific functional features are required during caries development (5, 10). Species of bacteria and fungi have been found to contribute to caries development, while not much is known about the contribution of other microorganisms (11). While no specific bacterial species or strain has been uniquely associated with caries development or established lesions, species of *Streptococci, Lactobacilli* and *Scardovia* are often found at high abundance (10). Although *Streptococcus mutans*, *Scardovia wiggsiae* and oral *Lactobacilli spp* have been shown to have a high inherent AT, other oral species often found during early biofilm formation have shown an ability to develop an acid tolerance response (ATR) when allowed to adapt to an acidic environment *in vitro* (12–14). The ATR has been proposed to involve upregulation of ATPases, alterations to the cell membrane making it less permeable to protons, and production of chaperones which protect bacterial DNA and proteins (15). The proteome of oral species has also been shown to be affected by the availability of sucrose and by environmental pH, with alterations visible in glycolysis, acid production and AT (16, 17).

While next-generation DNA sequencing methods have identified an increased abundance of acid-tolerant and acid-producing species during caries development, the functional properties of the overarching microbial community during caries activity need more exploration to understand the relationship to disease, and to find new biomarkers for disease prediction (8, 18). Biomarkers that have been used in dental practice to predict dental caries include microbial tests, saliva flow and previous caries experience, however, studies evaluating the accuracy of current caries risk assessment methods indicate that new prediction models are needed (19, 20). As the AT of oral strains is an important virulence factor during caries development, an increase in plaque AT could be a possible indication of caries activity at an early stage. The AT of specific bacterial species as well as of plaque from caries-active lesions has been studied extensively and a study of plaque harvested from the buccal and lingual smooth and intact sites of children aged 3–5 years, has demonstrated that *S. mutans* strains from caries-active children have a higher AT than those from caries-free children, although no differences in acidogenicity (ability to lower pH after a glucose pulse) were seen (21). Another investigation of the effect of a daily rinse of sucrose over one week in adolescents showed an increase in the number of bacteria able to grow at pH 5.5 (22), suggesting that an increased intake of dietary carbohydrates is reflected in the phenotype of the bacteria present in dental biofilms. However, to our knowledge, the AT on the microbial community level in young children with high caries activity has not been well studied.

Since increases in plaque AT are predicted to occur early in the caries process and have been shown to be stable over short periods of time, this phenotypic property of plaque could offer promise as a biomarker candidate for predicting caries (23). However, in order to test the potential of AT as a predictive biomarker it is first necessary to show that it is high in individuals with high caries activity and low or absent in healthy subjects. The aim of this study was therefore to determine whether the AT in children with severe caries is higher than in those with no caries activity. In addition to AT, the microbial composition of the plaque samples as well as differences in metabolic activity after a glucose pulse were also investigated to examine the relationship between specific bacterial species or acid production and AT.

## 2 Materials and methods

### 2.1 Selection of study subjects

Fifty children aged 2–5 years were included in the study. The number of participants required in each group to detect a 1 score difference in AT between groups based on a SD of 1.06 (20) was calculated as 18. Power was set to 80% with a 95% confidence interval. To allow for the possible need for exclusion of samples due to technical considerations, 25 individuals were included in each group.

The caries-active (CA) children were recruited at the Specialist clinic in Paediatric Dentistry, Faculty of Odontology at Malmö University, Malmö, Sweden to which they had been referred due to severe manifest caries in the primary dentition. Inclusion criteria were ≥3 decayed teeth (dt) (24), and high caries risk according to the regional risk assessment scheme for Region Scania, based on caries progression variables with a scoring range from Low/Medium/High. Clinical examinations were performed by one experienced specialist in paediatric dentistry, using optimal lighting, mirror and probe.

The caries-free (CF) group was recruited at the Public Dental Health clinic in Alvesta in the county of Kronoberg, Sweden. Inclusion criteria were no past caries experience (dt = 0) (24), and a low caries risk assessment based on the regional risk assessment scheme for Region Kronoberg. Clinical examinations were performed by one experienced general dentist, using optimal lighting, mirror and probe.

For both groups, children of Nordic and non-Nordic origin were included. Exclusion criteria were antibiotic treatment up to three months prior to sampling, use of medication, systemic or autoimmune disease or functional disorders deemed to affect oral health. No recommendations for oral hygiene or diet were given prior to sampling.

### 2.2 Ethics statement

This study was reviewed and approved by the Swedish Ethical Review Authority, reference 2020-01187 and 2021-03680. The participants legal guardian provided a written informed consent to participate in this study prior to sampling.

### 2.3 Sample collection

Sample collection was performed by the clinical examiner at the time of examination from January 2021 until December 2022. Plaque from all buccal and lingual surfaces of the upper and lower jaw, from both intact and decayed dental enamel and dentine was collected using a sterile plastic carver and pooled in an Eppendorf tube containing 500 µl of sterile UHQ-water. Arrival of the samples at the Section for Oral Biology and Pathology, Faculty of Odontology, Malmö University occurred within 24 h. The volumes of the samples were checked on arrival and adjusted when needed to 500 µl. The pooled plaque sample from each individual was then vortexed for 2 × 30 s and divided into three tubes. A 250 µl aliquot of the sample was stored at −80°C for 16S *r*RNA sequencing and another 150 μl aliquot was stored at −80°C for metabolite measurement (see below). The remaining 100 µl was used for measuring the AT of the plaque sample.

### 2.4 Acid tolerance assessment

Measurement of AT was performed within 24 h of arrival of the sample. The 100 µl aliquot was vortexed vigorously and divided into two Eppendorf tubes before being centrifuged for 5 min, 1,300 rpm (Eppendorf, Centrifuge 5415 D) at room temperature. The supernatant was discarded and 25 μl of TYE (Tryptone Yeast extract, 0.4M glucose, 0.4M Phosphate/Citrate buffer) adjusted to pH 3.5 for AT assessment or pH 7.5 (control for sample viability assessment) was added to the tubes. The sample was then mixed by pipetting and incubated aerobically for two hours at 37°C. LIVE/DEAD^®^ BacLight™ solution (Molecular Probes, Eugene, OR, USA) was then added according to manufacturer's instructions and the sample transferred to an Ibidi® μ-slide VI Ibi-treat flow-cell. The flow cells were gently centrifuged at 1,000 rpm for 60 s followed by examination with confocal laser scanning microscopy (CLSM) using a Nikon Eclipse TE2000 microscope (Nikon Corp., Tokyo, Japan) with an Ar laser (488 nm laser excitation). Images were acquired with a Photometrics Prime 95B camera using Nikon NIS-Elements software. Ten randomly selected images from each sample were saved for image analysis.

### 2.5 Image analysis of sample viability

Image analysis was performed on LIVE/DEAD^®^ BacLight™ stained samples, where bacteria with intact cell membranes fluoresce green and bacteria with compromised membranes fluoresce red. Sample quality after transfer from the clinic was assessed by incubation at pH 7.5 (control samples). Of the 50 plaque samples collected, only 42 controls showed more than 90% viability and 5 samples from the CA group and 3 samples from the CF group were therefore excluded from the study at this stage. Two additional samples from the CF group were excluded due to lack of material for AT analysis (<5% surface coverage in the confocal images).

### 2.6 Image analysis of acid tolerance

The remaining 40 plaque samples (20 from each group) were assessed for levels of AT after incubation at pH 3.5. The 10 images from each sample were assessed by two independent observers, using a previously validated AT scoring system ranging from 1 to 5 (23). Cells with intact cell membranes, as assessed after BacLight staining, were considered acid tolerant and cells with compromised cell membranes considered non-acid tolerant. All images were coded, so that the caries status was unknown to the observers. The mean score of each sample was included in the statistical analysis comparing the two groups, using the non-parametric Wilcoxon signed-rank test. *P*-values of less than 0.05 were considered statistically significant. For sample scores with an inter-observer-disagreement (none greater than 0.4), a mean value between the observers was calculated and included in the statistical analysis.

### 2.7 Induction of metabolic processes by glucose pulse

An aliquot (250 µl) of the suspended pooled plaque sample was diluted with 1.2% NaCl solution to a final concentration of 0.9% NaCl followed by vortexing and mixing through pipetting. Sterile glucose solution (1M) was added to the sample to give a final concentration of 20 mM glucose and the sample was incubated at 37°C for 60 min. After incubation, the sample was placed on ice for 5 min, before being centrifuged at 14,000 rpm for 10 min. The supernatant was transferred to a cryotube and stored at −80°C for NMR analysis.

### 2.8 Analysis of bacterial metabolites by nuclear magnetic resonance (NMR)

Samples were thawed at room temperature for 15 min, and centrifuged at 14,000 xg, 4°C, 10 min (Eppendorf 5804R, FA-45-30-11 rotor). The supernatant (585 µl) was transferred to a deep-well plate containing 65 µl buffer (400 mM potassium phosphate, pH 7.4, 1.548 mM 3-(trimethylsilyl)propionic-2,2,3,3-d4 acid (TSP-d4) and 0.13% w/v sodium azide) in each well. The samples were shaken at 12°C, 500 rpm for 2 min using a Thermomixer Comfort (Eppendorf) and then 575 µl of each sample was transferred to a 5 mm SampleJet NMR tube rack using a SamplePro Tube L liquid handling robot (Bruker BioSpin). ^1^H NMR data was collected on a 700 MHz Bruker Avance III spectrometer equipped with a 5 mm QCI cryoprobe and SampleJet sample exchanger. The standard Bruker pulse sequence “noesygppr1d” was used to collect 128 scans into 64 k data points, using a spectral width of 30 ppm, an acquisition time of 1.6 s, a relaxation delay of 4 s and a receiver gain setting of 181. Data was line broadened with an 0.3 Hz exponential before Fourier transformation. Spectra were referenced to TSP-d4. All spectral processing was done within TopSpin 3.6.2 (Bruker BioSpin). Metabolite concentrations were assessed with ChenomX 9.0 (ChenomX Inc.) using the TSP-d4 as internal standard.

### 2.9 DNA extraction

DNA from plaque samples was extracted as previously described (20). Briefly, 130 μl of lysis buffer combined with 10 μl of enzymatic cocktail (25 mg/ml of lysozyme (Appli chem, Cat. No A4972,0001), 1.25 KU/ml of lysostaphin (Sigma-Aldrich, Cat. No SAE0091-2MG), 0.625 KU/ml of mutanolisine (Sigma-Aldrich, Cat. No SAE0092-10KU) and 125 KU/ml of zymolase (Sigma-Aldrich, Cat. No SAE0098-20KU) was added and incubated at 37°C for 1 h. After that, 20 μl of glucanex 1% (10 min at 60°C) (Sigma-Aldrich, Cat. No L1412-5G) and proteinase K were added and incubated for an additional 15 min at 65°C followed by 10 min at 95°C. Then, DNA was isolated by MagNA Pure LC 2.0 Instrument (Roche Diagnostics, Risch-Rotkreuz, Switzerland), using the MagNA Pure LC DNA Isolation Kit III for Bacteria and Fungi (Roche Diagnostics, Cat. No. 03 264 785 001) following the manufacturer's instructions.

DNA concentration was estimated with the Qubit™ 3 Fluorometer (ThermoScientific) and then the V3-V4 hypervariable region of the 16S rRNA gene was amplified using the universal primers Forward (CCTACGGGNGGCWGCAG) and Reverse (GACTACHVGGGTATCTAATCC). A library was constructed using the Metagenomic Sequencing Library Preparation Illumina protocol (Part #15044223 Rev. A) and sequenced at the FISABIO Institute (Valencia, Spain) using 2 × 300 bp paired-end sequencing with an Illumina MiSeq instrument. Sequencing data have been publicly deposited in the SRA database (Bioproject PRJNA1157018).

### 2.10 Bioinformatic analysis

Sequencing output was analyzed as previously described using DADA2 R Statistics package (v1.20.0) (25). Briefly, reads trimmed by length were removed if they exceeded more than 5 expected errors, dereplicated and estimated sequencing errors using loessErrfun. R1 and R2 reads were merged (minimum overlapping region of 15 bp) after true sequence variants were inferred. After chimeric removal, ASVs were identified and annotated using SILVA v.138.1 database (26). In order to calculate statistical differences in the abundance of taxons between groups, the data was transformed using ANCOMBC2 (27) approach and then compared using paired non-parametric Wilcoxon test (wilcox.test function of stats library of R) (28). Adjusted p-values were calculated using false discovery rate. Correlations between metabolites concentration and bacterial proportions were calculated using the mixOmics package. For both ANCOMBC and correlations, data was filtered by low abundant and prevalent species. Finally, for rarefaction curves, richness and diversity analyses at the species level we used the minimum number of reads annotated in a sample at the species level (4.5 × 10^5^).

## 3 Results

### 3.1 Characteristics of study population

The mean age of the participants was 4.5 years (±0.97) in the CA group (13 male and 7 female), and 4.7 years (±0.57) in the CF group (11 male and 9 female), with no statistically significant differences between the two groups. The mean number of decayed teeth at the time of sampling was 0 for the CF and 8.0 (±3.2) for the CA group (Table 1).

**Table 1:** Characteristics of the CA group regarding number of decayed teeth (dt).

*N*	Valid	20
	Missing	0
Mean		8.0
Median		7.0
Std. deviation		3.2
Minimum		3
Maximum		15

Number of decayed teeth ranged between 3 and 15 with a mean value of 8.0 (±3.2).

### 3.2 Acid tolerance (AT)

**Figure 1:**
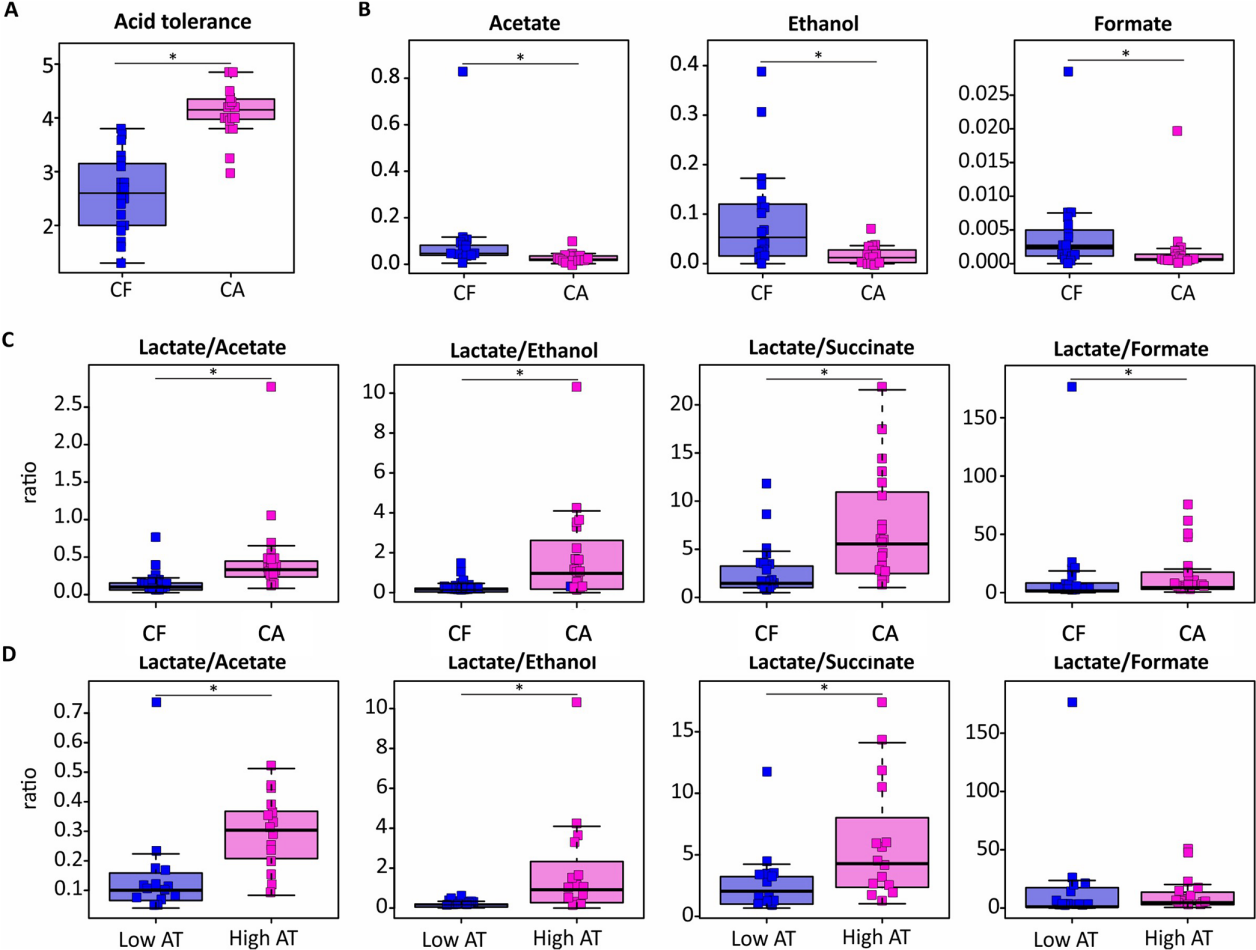
Effect of severe caries on the AT and the concentration of dental plaque-derived metabolites after a 20 mM glucose pulse. Boxplots in caries-active (CA) and caries-free (CF) groups represent acid tolerance values **(A)**, metabolite concentrations (mM) **(B)**, and ratios of lactate production against other organic acids **(C)**. The differences between plaque samples with a low AT score (Score 1–2) and a high AT score (score 4–5) are also included **(D)**.

Assessment on arrival at the laboratory revealed a viability of over 90% in all plaque samples included for further analysis. The AT was evaluated by assessing the integrity of the bacterial membranes following an acid challenge of pH 3.5 and scoring each sample using a validated scoring table ranging from 1 (low AT) to 5 (high AT) (23). The AT scores of the CA samples ranged between 3.00–4.85 while those of the CF samples ranged between 1.3–3.8. Interestingly, the mean AT score of the CA group (4.1) was significantly higher (adjusted *p* = 8 × 10^−6^) than the mean AT score of the CF group (2.6) (Figure 1A). In addition, the samples with the highest AT scores (score 4.0–5.0) all belonged to the CA group, representing 75% of the group, while the low AT scores (score 1.0–2.0) all belonged to the CF group, representing 70% of the group.

### 3.3 Organic acid production

To further explore the phenotypical traits of the plaque samples, the metabolic activity was investigated after a 20 mM glucose pulse for one hour. While a total of 23 metabolites were detected by NMR in the sample supernatant, the known end-products of glucose metabolism identified were acetate, formate, propionate, succinate, ethanol, lactate and butyrate. When comparing the two groups, the production of acetate, formate, and ethanol was significantly higher in the CF group compared to the CA group (Figure 1B). No significant difference in lactate production between the two groups was observed, although, when comparing the ratios of lactate against the other products of glucose metabolism, the ratio of lactate to formate, succinate, acetate and ethanol was significantly higher in the CA group compared to the CF group (Figure 1C). The ratio of lactate to succinate, acetate and ethanol was also higher in the samples with a high AT (Score 4–5) compared to those with a low AT (Score 1–2), while no significant difference in the ratio of lactate to formate was observed (Figure 1D).

### 3.4 Bacterial composition

The mean number of sequences obtained per sample was 295,000, with the rarefaction curves showing that the diversity was fully covered at the species level after 114,000 reads (Supplementary Figure S1). When comparing the composition of the plaque samples of the two groups at genus level, similarities were seen in the genera represented. Streptococci were well represented in both groups and other genera with a high relative abundance were *Leptotrichia, Neisseria, Capnocytophaga* and *Actinomyces* (Supplementary Figure S2).

**Figure 2:**
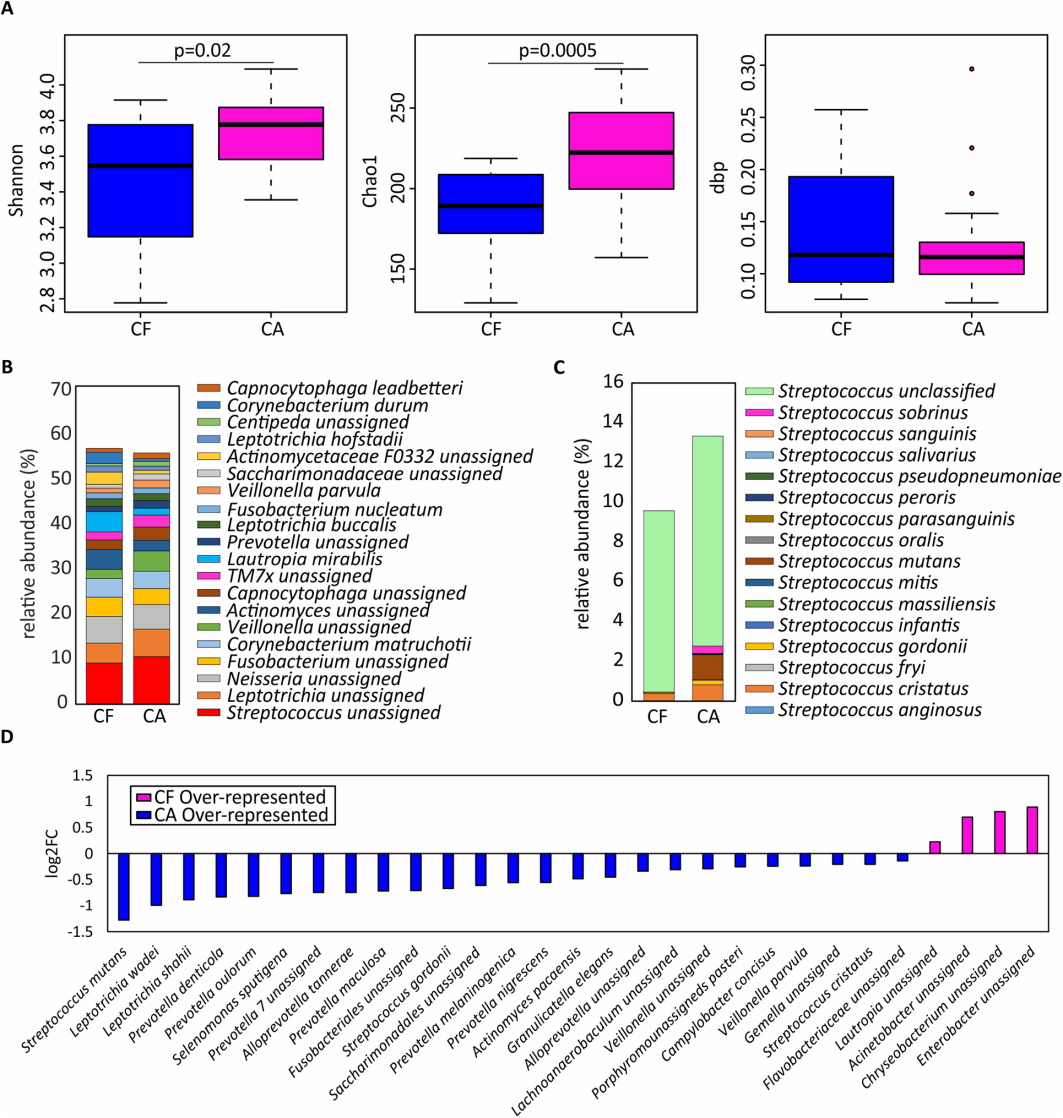
Microbial variations in dental plaque from children affected by severe caries. **(A)** Shannon diversity index, Chao1 richness index and dbp dominance index are shown as boxplots in children with severe caries (CA) and those who were caries-free (CF). **(B)** Relative abundance of the top-20 most abundant species. **(C)** Relative abundance of bacterial species classified within the *Streptococcus* genus. **(D)** Bacterial species differentially represented in CF and CA individuals, expressed as log_2_ fold change (log_2_FC).

At the species level, sample richness and diversity were significantly higher in the CA group compared to the CF group, while no significant difference in dominance was seen between the two groups (Chao1 index, *p* = 0.00023; Shannon index, *p* = 0.024; see Figure 2A). In both groups, a high relative abundance of unassigned species of *Streptococcus, Leptotrichia, Neisseria* and *Fusobacterium* was observed, while *Corynebacterium matruchotii* and *Lautropia mirabilis* had the highest relative abundance among the assigned species (Figure 2B). When comparing the abundance of species included in the *Streptococcus* genus between the two groups, *Streptococcus cristatus* had the highest relative abundance of the streptococci identified in the CF group (Figure 2B), while *S. cristatus*, *S. mutans* and *Streptococcus sobrinus* had the highest relative abundance in the CA group (Figure 2C).

When including species with a relative abundance greater than 0.1%, 25 species were shown to be overrepresented in the CA samples (Figure 2D). Among these, *S. mutans* was the most dominant species in the CA group and was detected in 95% of the samples, compared to 30% of the samples in the CF group (data not shown). Other species often associated with caries which were found to be overrepresented in the CA group were *Veillonella parvula* as well as two species of *Leiptotrichia (L. wadei* and *L. shahii)*. Interestingly, several species of the proteolytic and anaerobic genus *Prevotella* (*P. denticola, P. oulorum, P. maculosa, P. melaninogenica* and *P. nigrescens*) also showed a significantly higher relative abundance in the CA group. A higher relative abundance of *Scardovia wiggsiae* was also observed in the CA group compared to the CF group, although the difference did not reach significance at the 5% level.

In order to discern whether certain species were associated with high or low AT, a correlation analysis was performed between bacterial abundance and AT score. Surprisingly, the species with the strongest positive correlation with AT in the CA group was an unassigned species of *Porphyromonas*, as well as *Porphyromonas pasteri*. Meanwhile in the CF group, unassigned species of *Leptotrichia, Fusobacterium* and *Tannerella* as well as *Lachnoanaerobaculum sabbureum* and *Corynebacterium matruchotii* were negatively correlated with AT whereas unclassified *Rothia, Neisseria* and *Streptococcus* species were positively correlated with AT in CF individuals.

**Figure 3:**
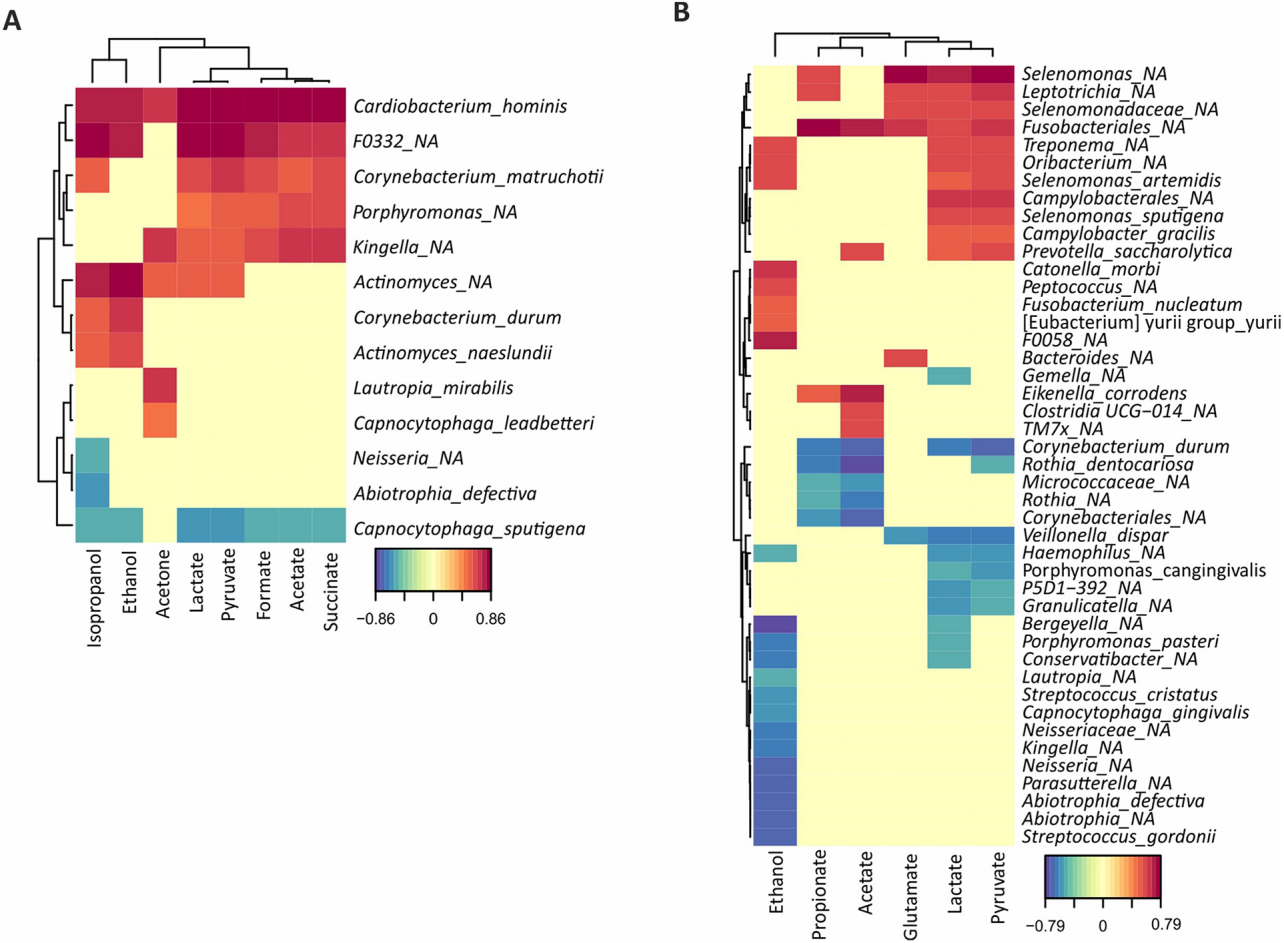
Correlation analysis of bacterial abundances with metabolite concentrations quantified by NMR after a 20 mM glucose pulse. The degree of positive (red) or negative (blue) correlation are indicated in the legend for both caries-free **(A)** and caries-active **(B)** individuals.

Surprisingly, when correlating individual metabolites to the levels of different species, unidentified genera of the *Fusobacteriales* order showed the strongest association to the production of lactate, propionate, acetate and pyruvate in the CA group while a negative association for the same metabolites was seen for the species *Corynebacterium durum* (Figure 3B). Meanwhile, in the CF group, the species with the strongest association to acid production was instead *Cardiobacterium hominis,* whereas *Capnocytophaga sputigena* showed a negative association with the acidic end-products (Figure 3A).

## 4 Discussion

Consistent with the idea that an acid tolerant microflora is required for caries development, a higher AT was observed in the CA group compared to the CF group. This might partly be related to the increased abundance of inherently acid tolerant strains such as *S. mutans* in the increasingly acidic environment, as well as an adaptation of other oral strains to the acidic environment developed during caries progression (14). Caries development is a dynamic process with numerous bacterial properties contributing to each stage, where acid tolerance genes have been suggested to be mainly involved in the earlier stages of caries development, while genes for osmotic stress and proteases seem to be overrepresented in dentinal caries (29). Since plaque from each individual subject was pooled, functional properties that might have been displayed if communities at different stages of caries activity had been analyzed, could not be assessed in this study. However, the advantage of pooling samples is that it allowed for analysis of plaque from all stages of caries development together with sites without visible signs of caries activity in the CA group, where the overall AT was higher than in the CF group. This differs to the findings of Havsed et al. (20), who examined the AT of plaque from adolescents with varying degrees of caries, where no significant differences in the AT between CA and CF individuals was identified, although 7 out of 10 individuals with the highest AT scores belonged to the CA group.

Production of lactate as an end-product of glucose metabolism by oral strains is regarded as a key factor in promoting low pH in caries-associated biofilms (30). To investigate the metabolic activity and whether plaque from CA children generated more lactate than that from CF children, we analyzed the levels of metabolites present in the supernatant of glucose-pulsed samples. Based on the results from the present study, the production of ethanol, acetate and formate was significantly higher in the CF group compared to the CA group while no significant differences were observed in the production of other organic acids. When comparing the ratio of lactate to acetate, ethanol, formate or succinate, these were higher in the CA group compared to the CF group. These results are in line with those of Havsed et al. (20), where they were not able to see a difference in the production of specific acids, but the ratio of lactate against other organic acids was higher in the CA group.

Unidentified species of *Fusobacteriales* order in the CA group showed a strong association to lactate as well as propionate, acetate and pyruvate. Although species such as *Fusobacterium nucleatum* can metabolize glucose with a resultant lowering of the environmental pH *in vitro* (31), the association to organic acid production observed in this study is probably more related to the properties of the plaque communities where these species are found, rather than specifically reflective of the properties of these species. While often present in healthy plaque and sites of periodontal lesions, *Leptotrichia spp* as well as *Fusobacterium spp* can adhere to a large number of oral species during biofilm formation and is therefore often found in mature plaque as well as all stages of caries development (32, 33).

Even though *Veillonella* has been shown to produce acetate and succinate from lactate, and this genus had a higher relative abundance in the CA group, this was not matched by significantly higher levels of succinate or acetate in this group. This might be due to other known lactate consumers present in both groups, such as *Actinomyces, Prevotella* and *Neisseria* (5, 8, 13, 34). In this study, the handling of the plaque samples was performed under aerobic conditions. Although many oral strains can metabolize glucose in an aerobic environment, a minor difference in the end-products could have been observed if handled anaerobically as the metabolic pathways used by the bacteria might differ (8).

The diverse dental microbiota in both CA and CF individuals displayed differences regarding both the relative abundance of species as well as the phenotypical traits of the biofilms. As the collected samples were pooled from all buccal and lingual surfaces, the functional properties observed can be attributed to the total dental microbiome of the individuals. These results support the current hypothesis that dental caries is not induced by single pathogenic species but a complex biofilm community, which drives ecological changes that affect both biofilm composition and its functional properties (9, 10, 35).

While similarities were observed in the species represented, the relative abundance of these differed between the two groups. As expected, *S. mutans* was highly over-represented in the CA samples, which is consistent with other studies on the microbial composition of plaque from caries-active individuals (36, 37). *Streptococcus mutans* has been shown to be present in approximately 85% of individuals with active caries, with the numbers varying depending on population and sampling site (35, 36), and a high relative abundance of *S. mutans* might be an indication of an acidic biofilm environment (38). As seen in other studies, we observed *S. mutans* in CF individuals although the prevalence and relative abundance were significantly lower than in CA individuals. Another highly saccharolytic species, *Scardovia wiggsiae* was found at higher abundance in the CA group, although the difference observed was not statistically significant. *S. wiggsiae* has previously been associated with caries in both permanent and deciduous teeth and its high saccharolytic activity at low pH increases its competitiveness in an acidic environment (38). In agreement with Havsed et al. (20), a higher abundance of *Veillonella spp* was found in the CA group compared to the CF group. Since *Veillonella spp* are able to utilize organic acids as a carbon source (39), and its combined growth with *S. mutans* increases the final output of organic acids (40), the high abundance of this species might be a marker of an increased presence of organic acids during caries development. The high abundance of *L. shahii* and *L. wadei* observed in the CA group seem to be in accordance with other studies investigating the composition of plaque from CA children (41). Due to these species ability to produce lactic acid from carbohydrates, they might contribute to the low pH driving the demineralization of dental hard tissues. The surprisingly large number of *Prevotella* species found in the CA group could be the result of the severe caries activity of the study subjects. Plaque samples were obtained from both healthy and CA sites, where dentinal lesions were included. Species of *Prevotella* are highly proteolytic anaerobes and have often been associated with periodontitis. On the contrary, they have also been found in large numbers in dentinal cavities (41–43). Their presence in dentine lesions might be due to the high organic content of dentin and anerobic environment of deep lesions, although, the saccharolytic to moderately saccharolytic properties of these species might also play a role in the demineralization of dental hard tissues (29, 44, 45). In a longitudinal study investigating pooled plaque samples of children aged 1–6, a domination of proteolytic taxa such as *Prevotella* was already detected 1–3 years prior to the clinical detection of dental caries (37). Species that have been associated with a healthy plaque in the primary dentition in other studies, such as *Neisseria spp* (17, 46) was not found in a higher abundance in the CF group in this study, which might be due to the samples being collected at the time that consent to participate in the study was given. Refraining from oral hygiene measures 24 h before sampling might have led to a more mature biofilm sample from the CF group.

The results of this study support the idea that complex interactions take place during caries development, promoting changes in the microbial composition which contribute to changes in the phenotypical properties of the biofilm. A higher abundance of both saccharolytic species as well as proteolytic anaerobes was identified in the caries-active children and the metabolic end-products after a glucose pulse indicate differences in the metabolic pathways used. The AT of dental plaque, as assessed as viability after an acid challenge, is significantly higher in children with high caries activity compared to caries-free children. Analysis of the *phenotype* of dental plaque as a whole might therefore serve as a good indication of the caries status of individuals. However, longitudinal studies investigating how the AT changes over time in relation to caries development are needed before plaque AT can be considered as a prediction method for the development of dental caries.

## Data Availability

Original datasets are available in a publicly accessible repository: The original contributions presented in the study are publicly available. This data can be found here: https://www.ncbi.nlm.nih.gov/bioproject/ (Project number: PRJNA1157018).
