# Supplementary material for: Oral biofilm composition and phenotype in caries-active and caries-free children
Source: Front Oral Health. 2024 Oct 22;5:1475361. doi: 10.3389/froh.2024.1475361 (PMC11534697; doi:10.3389/froh.2024.1475361)
Supplement: Supplementary file 1 [file Image1.pdf]

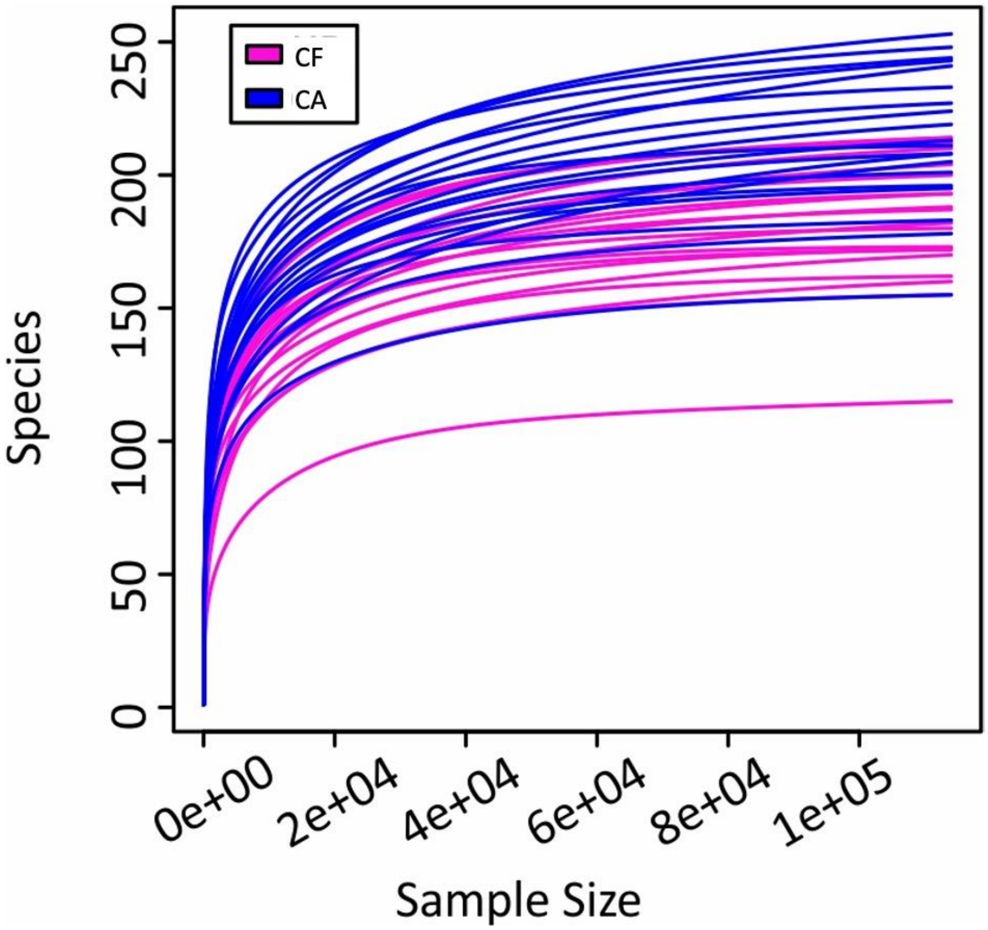

Supplementary Figure 1. Bacterial richness coverage by the Illumina sequencing strategy. The rarefaction curves show the estimated number of bacterial species detected in relation to the number of reads obtained (sequencing effort) for each sample in both studied groups.
