# Supplementary material for: Oral biofilm composition and phenotype in caries-active and caries-free children
Source: Front Oral Health. 2024 Oct 22;5:1475361. doi: 10.3389/froh.2024.1475361 (PMC11534697; doi:10.3389/froh.2024.1475361)
Supplement: Supplementary file 2 [file Image2.pdf]

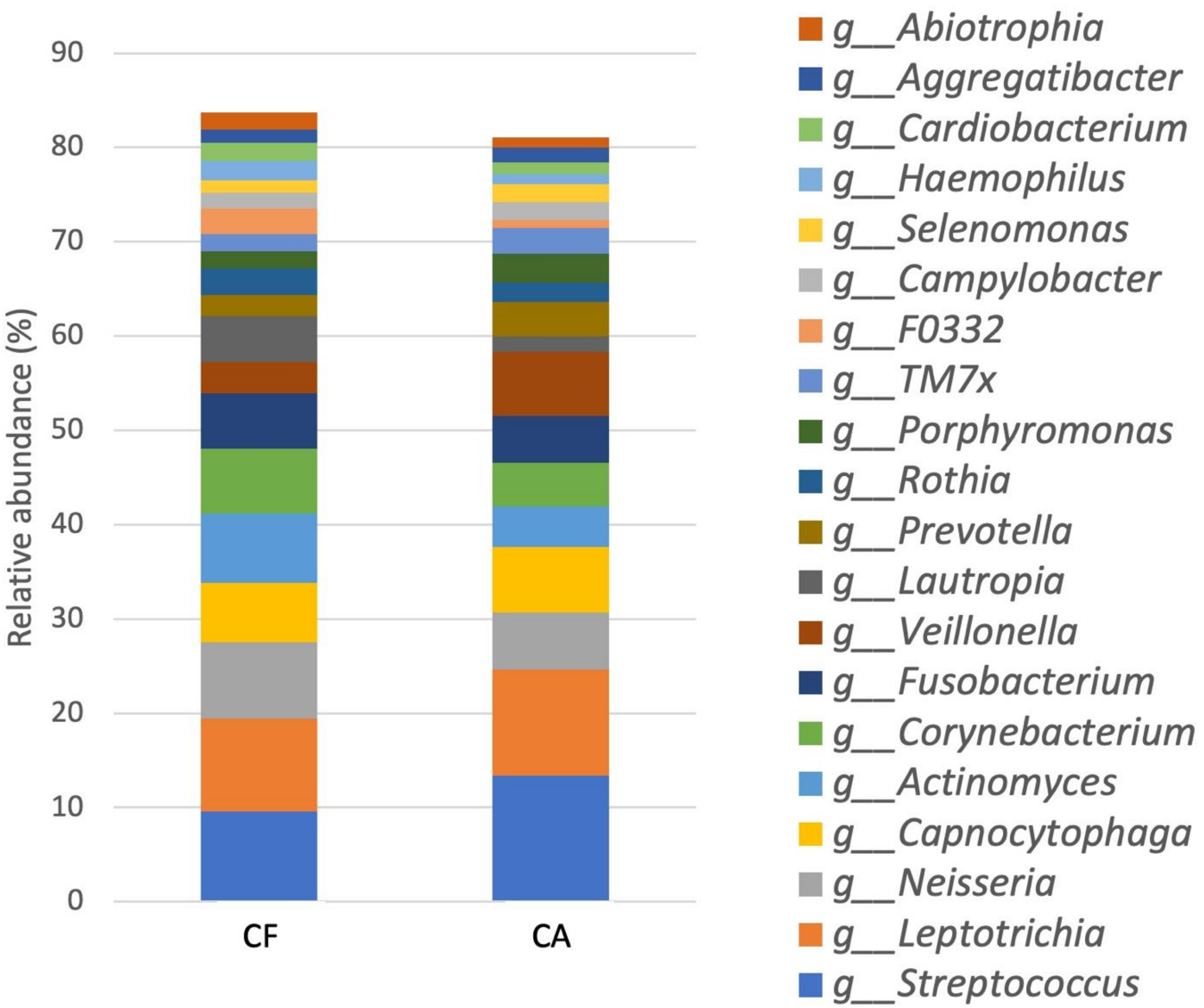

Supplementary Figure 2. Composition of bacterial plaque samples from caries-free (CF) and caries-active (CA) individuals displayed as relative abundance of bacterial genera based on 16S rRNA Illumina sequencing.
